# Heparin-free veno-venous extracorporeal membrane oxygenation in a multiple trauma patient: A case report

**DOI:** 10.1097/MD.0000000000019070

**Published:** 2020-01-31

**Authors:** Youn Young Lee, Hee Jung Baik, Heeseung Lee, Chi Hyo Kim, Rack Kyung Chung, Jong In Han, Hyunyoung Joo, Jae Hee Woo

**Affiliations:** a Department of Anesthesiology and Pain Medicine, Ewha Womans University Hospital; b Department of Anesthesiology and Pain Medicine, College of Medicine, Ewha Womans University, Seoul, South Korea.

**Keywords:** acute respiratory failure, extracorporeal membrane oxygenation, heparin, trauma

## Abstract

**Rationale:**

Extracorporeal membrane oxygenation (ECMO) in multiple trauma patients with post-traumatic respiratory failure can be quite challenging because of the need for systemic anticoagulation, which may lead to excessive bleeding. In the last decade, there is a growing body of evidence that veno-venous ECMO (VV-ECMO) is lifesaving in multiple trauma patients with acute respiratory distress syndrome, thanks to technical improvements in ECMO devices.

**Patient concerns:**

We report a case of a 17-year-old multiple trauma patient who was drunken and had confused mentality.

**Diagnoses:**

She was suffered from critical respiratory failure (life-threatening hypoxemia and severe hypercapnia/acidosis lasting for 70 minutes) accompanied by cardiac arrest and trauma-induced coagulopathy during general anesthesia.

**Interventions:**

We decided to start heparin-free VV-ECMO after cardiac arrest considering risk of hemorrhage.

**Outcomes:**

She survived with no neurologic sequelae after immediate treatment with heparin-free VV-ECMO.

**Lessons:**

Heparin-free VV-ECMO can be used as a resuscitative therapy in multiple trauma patients with critical respiratory failure accompanied by coagulopathy. Even in cases in which life-threatening hypoxemia and severe hypercapnia/acidosis last for >1 hours during CPR for cardiac arrest, VV-ECMO could be considered a potential lifesaving treatment.

## 1 Introduction

The use of extracorporeal membrane oxygenation (ECMO) in multiple trauma patients with refractory respiratory failure has been suggested as salvage therapy that can improve outcomes.^[1–3]^ However, the clinical benefit of ECMO support in critical trauma patients remains unclear. Careful consideration should be given to the risks and benefits before starting ECMO support because of the risk of organ bleeding, especially in multiple trauma patients. Heparin-free VV-ECMO could be an effective treatment strategy in trauma patients with contraindications to systemic anticoagulation, including multiple trauma patients with traumatic brain injury.^[4]^ We report a case of a 17-year-old multiple trauma patient who suffered from critical respiratory failure with life-threatening hypoxemia and severe hypercapnia/acidosis lasting for 70 minutes accompanied by cardiac arrest and trauma-induced coagulopathy during general anesthesia. She survived with no neurologic sequelae after immediate treatment with heparin-free VV-ECMO. The patient had provided informed consent for publication of this case report.

## 2 Case presentation

A 17-year-old girl, 160 cm in height and weighing 48 kg (BMI, 18.75 kg/m^2^) came to the emergency room (ER) due to a motorcycle accident. She was drunk and had a confused mental state. She had multiple fractures in her left femur, right pelvis, both mandibles, multiple right ribs (1–5^th^), and tooth #21, and severe right lung contusion with several lacerations on the scalp. Her injury severity score (ISS) was 34 points, representing a severe grade of injury.

Her initial vital signs and laboratory findings were as follows: blood pressure (BP), 98/60 mm Hg; heart rate (HR), 118 beats/min; respiratory rate (RR), 38/minute; saturation on pulse oximetry (SpO_2_), 98% in room air; hemoglobin (Hb), 10.1 g/dL; hematocrit (Hct), 30.6%; platelets, 385,000; prothrombin time (PT), 14.4 seconds; INR, 1.27; and activated partial thromboplastin time (aPTT), 29.1 seconds. Five minutes after she entered the ER, her SpO_2_ suddenly dropped to 85%, and she was intubated immediately and mechanically ventilated under pressure-regulated volume-controlled (PRVC) mode {tidal volume (*V*_T_), 350 mL; inspired oxygen fraction (F_I_O_2_), 0.4; RR, 14 breaths/min; positive end-expiratory pressure (PEEP), 7 cm H_2_O}, and a right-sided chest tube was inserted.

**Figure 1:**
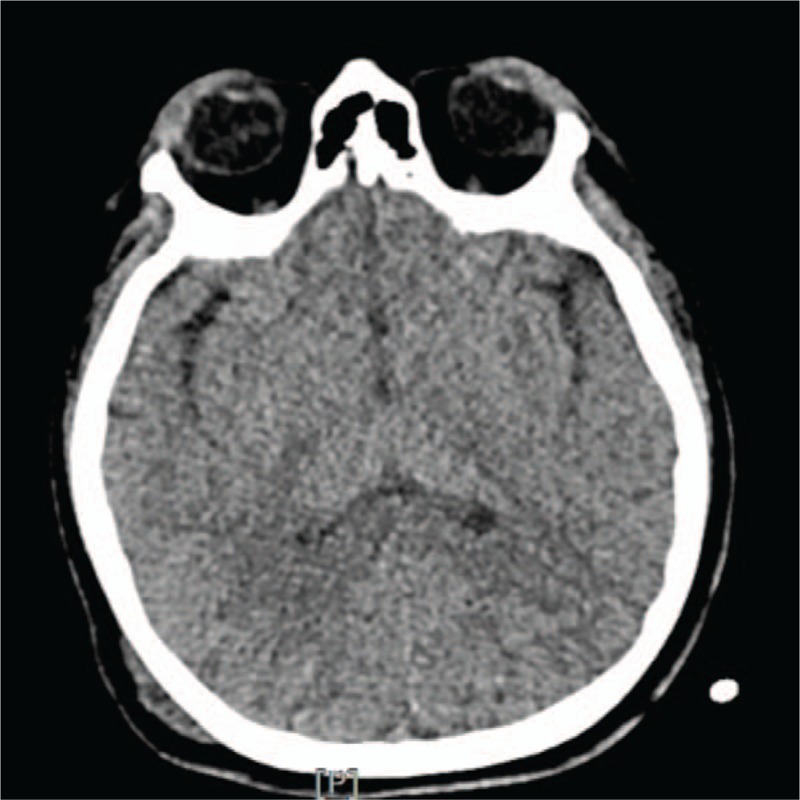
CT of the brain. There are hematoma and emphysema along the right frontal, parietal scalp and both occipital scalp area, but there is no evidence of intracranial hemorrhage. CT = computed tomography (CT).

**Figure 2:**
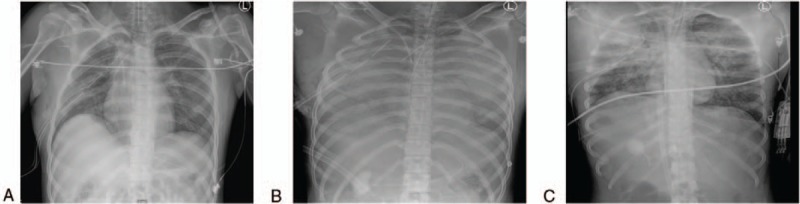
Chest images. (A) Preoperative chest PA (7 h before anesthesia), (B) intraoperative chest AP (5 min before ECMO), and (C) postoperative chest AP (3 h after ECMO). ECMO = extracorporeal membrane oxygenation.

Catheters were inserted into both her subclavian veins for active fluid resuscitation and blood transfusion. Even though she was given 2000 mL of crystalloid fluid and 5 units of packed red blood cells (pRBCs) for an hour in the ER, her BP suddenly dropped to 66/39 mm Hg and her HR increased to 130 beats/min; the ER physicians decided to administer 0.1 μg/kg/min norepinephrine. At the same time, her Hb, Hct, and platelet levels decreased to 6.8 g/dL, 20.5%, and 110,000, respectively. She was transfused with an additional 5000 mL of crystalloid fluid, 5 units of pRBCs, and 10 units of fresh frozen plasma (FFP), and was evaluated via CT of the brain (Fig. 1), abdomen and pelvis, facial bone, and chest PA (Fig. 2(A)). The ER physicians decided that she should undergo emergency surgical procedures on her left supracondylar femur fracture and both mandible fractures.

She arrived at the operating room (OR) in an intubated state with continuous infusion of 0.2 μg/kg/min norepinephrine; we immediately applied ventilation under volume-controlled (VC) mode {*V*_T_, 550 mL; F_I_O_2_, 1.0; RR, 15 breaths/min; PEEP, 5 cm H_2_O}. Her left radial artery was cannulated for arterial BP monitoring. Initial BP, HR, SpO_2_, and bispectral index (BIS) were 126/70 mm Hg, 120 beats/min, 100%, and 45, respectively. Fifteen minutes after the induction of total intravenous anesthesia using target effect-site concentration (EC)-controlled infusion (EC of propofol, 1.5 μg/mL; EC of remifentanil, 2 ng/mL), the surgery was started.

Twenty-five minutes after the start of surgery, her BP suddenly dropped from 130/100 to 78/40 despite a high dose of norepinephrine (3 μg/kg/min), her HR decreased from 151 to 90 beats/min, and her SpO_2_ to 75%. Peak inspiratory airway pressure (PIP) suddenly increased from 28 to 35 mm Hg with a large amount of pink frothy watery secretion discharged from the endotracheal tube (ET). We ventilated her lungs manually with frequent endotracheal suction, and her SpO_2_ was 52%. Consecutively severe bradycardia (40 beats/min) followed by cardiac arrest occurred; cardiopulmonary resuscitation (CPR) was started and a 1-mg epinephrine bolus was given five times during a 45-minute CPR. Severe hypoxemia, hypercapnia, and acidosis developed (Table 1). After the 45-minute CPR, return of spontaneous circulation (ROSC) was achieved and a chest X-ray was taken (Fig. 2(B)), which showed increased haziness in both lungs suggesting pulmonary edema, consistent with the large amount (2.4 L/1.5 hours) of pink frothy watery secretion from the ET even after ROSC.

**Table 1:**
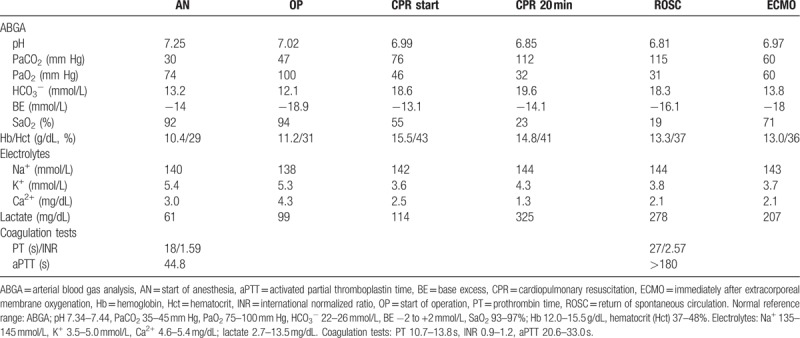
Laboratory findings of the patient in operating room.

We decided to start VV-ECMO therapy because of acute refractory lung failure and impending cardiac arrest even after ROSC (Table 1). We decided not to give bolus heparin considering the risk of hemorrhage. Her activated coagulation time (ACT) was 510 seconds and other coagulation tests showed prolonged times (Table 1). VV-ECMO (PLS-Set and Quadrox PLS; Maquet Cardiopulmonary AG, Rastatt, Germany) was applied on the right femoral vein, with a 19-Fr access cannula positioned 10 cm lower than the return cannula (21 Fr) by a cardiosurgeon. As the ECMO flow started, her severe hypoxemia and hypercapnia began to improve, as shown in Table 1. Even though we administered 20 mg furosemide twice, her urine output was <0.5 mL/kg/hour and the estimated blood loss was <30 mL. The surgery was discontinued and the patient was transferred to the intensive care unit (ICU); ECMO flow was 8.95 L/min with FiO_2_ of 1.0 and gas flow of 4 L/min; continuous renal replacement therapy (CRRT) was immediately started.

Three days after she entered the ICU, the ECMO flow rate gradually decreased to 3.19 L/min, with FiO_2_ of 0.21 under adaptive support ventilation (ASV) mode {F_I_O_2_ 0.3, *V*_T_ 250 ml, PEEP 16 cm H_2_O}. Her vital signs were stable without any inotropics and arterial blood gas analysis (ABGA) values showed a pH 7.382, PCO_2_ 44.1, PO_2_ 94.9, and SaO_2_ 97.1%. We decided to discontinue the VV-ECMO. Ventilator care was stopped on postoperative day (POD) 7, and 7L T-piece oxygen was started. On POD 10, we decided to stop CRRT and apply 2 L/min O_2_ via nasal prong, and her mental status improved from drowsy to alert.

During the 33 days of ICU care, she underwent three additional surgeries under general anesthesia. No intracranial hemorrhage was seen in serial brain CT, and she was discharged with no neurologic complications after 128 days of hospitalization.

## 3 Discussion

Although the technique of ECMO is highly invasive, it has been shown to be a lifesaving therapy in non-traumatic and traumatic respiratory failure with refractory hypoxemia that persists despite conventional treatment.^[2,3,5]^ The use of ECMO in multiple trauma patients may be challenging considering the risk of hemorrhage, especially in cases of severe coagulopathy, contraindications to anticoagulant use, or traumatic brain injury. Nevertheless, recent growing evidence suggests that applying ECMO as rescue therapy in trauma patients with severe respiratory failure may provide potential survival benefits that overweigh the associated risk of bleeding.^[2,6,7]^ Recent advances in ECMO devices and the application of heparin-free ECMO have dramatically increased safety profiles and expanded the application of ECMO to severe multiple trauma patients with coexisting bleeding shock or even traumatic brain injury.^[4,8]^

Our multiple trauma patient, who suffered from severe hypoxemia (31 mm Hg PaO_2_ at F_I_O_2_ 1.0) and hypercarbia (115 mm Hg) accompanied by cardiac arrest due to critical respiratory failure, survived due to application of heparin-free VV-ECMO.

Due to the risk of clot formation in patients on ECMO, anticoagulation is necessary for the prevention of thrombosis while avoiding excessive bleeding. Although anticoagulation in VV-ECMO is controlled by an ACT of 160 to 220 seconds and aPTT of 50 to 80 seconds in general,^[9]^ aPTT of 40 to 55 seconds may be sufficient to maintain modern VV-ECMO using heparin-bonded tubing.^[10]^ We decided not to give her anticoagulants during or after insertion of the ECMO circuit because coagulation tests before induction of anesthesia indicated prolonged times (PT, 18 seconds; INR, 1.59; aPTT, 44.8 seconds) and her platelet count was 84,000, indicating that trauma-induced coagulopathy (TIC) had developed. TIC, which is acute intrinsic coagulopathy arising in severely injured trauma patients, occurs in the presence of both tissue hypoperfusion due to hemorrhage and severe anatomical tissue injury.^[11]^ She was intubated and received a massive blood transfusion and fluid resuscitation with vasopressor therapy due to hemorrhagic shock and severe hypoxemia in the ER, which was the likely cause of TIC. Immediately after ECMO started, her coagulation tests indicated more prolonged times (ACT, 510 seconds; PT, 27 seconds; INR, 2.59; and aPTT > 180 seconds). Her prolonged PT, INR, and aPTT, which were monitored daily, were sustained during (94 hours) and 4 hours after ECMO (PT, 16 seconds; INR, 1.41; and aPTT, 51.9seconds). Therefore, there was no need to give her anticoagulants during the ECMO.

Indications for VV-ECMO in acute respiratory failure include hypoxemic and/or hypercarbic respiratory failure, respiratory failure in lung transplantation, bronchopleural fistulas and pulmonary air leaks, and complex airway management.^[9]^ The most common indication for ECMO in respiratory failure is severe acute respiratory distress syndrome (ARDS).^[12]^ It has been reported that 4.6% of trauma patients develop ARDS and independent predictors for ARDS include injury severity, thoracic injury, polytrauma, pneumonia, and receiving more than 5 units of FFP and 6 to 10 units of pRBC, especially during early (first 24 hours) transfusion of pRBC.^[13]^ Although the cause of acute severe respiratory failure in our patient has not been fully elucidated and may involve transfusion-related acute lung injury, severe pulmonary edema or hemorrhage, or trauma-induced ARDS, she had many risk factors for traumatic ARDS including high ISS (34 points), polytrauma, severe right lung contusion, receiving early transfusion of pRBC (10 units within 8 hours) and receiving 10 units of FFP in the ER due to severe shock. Various triage criteria for ECMO in hypoxic and hypercarbic respiratory failure have been reported as follows:

(1) PaO_2_/F_I_O_2_ (P/F ratio) < 100 with F_I_O_2_ > 90% despite optimal care for 6 hours or more,^[14]^
(2) P/F ratio of <80 with PEEP 15–20 cm H_2_O, hypercarbia with high plateau pressures >30 cm H_2_O and a pH of 7.15,^[15]^
(3) P/F ratio of <50 with F_I_O_2_ > 80% for >3 hours, or
(4) P/F ratio < 80 for 6 hours with pH < 7.25 for 6 hours despite optimum mechanical ventilation.^[9]^

However, these criteria are applicable to ARDS patents in the ICU. In our case, cardiac arrest occurred due to severe acute respiratory failure with massive pulmonary edema during general anesthesia. Despite ROSC after a 45-minute CPR, severe hypoxemia (31 mm Hg PaO_2_), hypercapnia (115 mm Hg PaCO_2_), and acidosis (pH 6.81) continued with a continuous large amount of pink frothy watery secretion from the ET, preventing us from ventilating her lungs due to the need for frequent suction. Therefore, we applied VV-ECMO as a lifesaving treatment to prevent recurrence of cardiac arrest. This is the last choice in such an impending cardiac arrest situation. Thus, we consider that VV-ECMO in this patient was similar to extracorporeal cardiopulmonary resuscitation (ECPR) in spite of its application after ROSC. ECPR is a method of CPR using ECMO to support circulation in refractory cardiac arrest patients.^[16,17]^ Despite inconclusive evidence of the role of ECPR in cardiac arrest,^[16]^ there is a report of patient survival with good neurologic outcomes due to ECPR applied after an 80-minute CPR.^[17]^

Absolute contraindication to ECMO in respiratory failure is the presence of severe irreversible respiratory failure, and relative contraindications include prolonged use of high-pressure ventilation or high F_I_O_2_, limited vascular access, contraindications to the use of anticoagulation, the presence of disease or organ dysfunction (e.g., severe irreversible brain injury or untreatable metastatic cancer), inability to receive blood products, high-body mass index > 45, and major immunosuppression.^[9,12]^

Neurological complications including intracranial bleeding, brain death, and ischemic stroke in VV-ECMO could be critical and major causes of death.^[18]^ Improvements in the ECMO technique have reduced the incidence of neurological complications to approximately 7%.^[18,19]^ The pre-ECMO risk factors for neurological complications during VV-ECMO have been reported to be cardiac arrest, CRRT, and hyperbilirubinemia.^[18]^ Two different mechanisms of neurological injury may be involved in VV-ECMO. One is preexisting neurological injury before ECMO including anoxic brain injury or hypoperfusion-related brain injury. The other is anticoagulation.^[18]^ In the decision to start ECMO in our patient, life-threatening hypoxemia (PaO_2_, 31–46 mm Hg) and severe hypercapnia (PaCO_2_, 76–115 mm Hg)/acidosis (pH 6.81–6.99) lasting for 70 minutes and the 45-minute CPR were thought to be risk factors for neurological complications during VV-ECMO. Our minimal knowledge of the patient's neurological status due to her drunken state despite absence of intracranial hemorrhage in her initial brain CT, the episode of hypoxemia with hemorrhagic shock in the ER, TIC, or CRRT in the ICU were also possible risk factors. Fortunately, she recovered with no neurological complications after three days of ECMO therapy and three surgeries under general anesthesia. Her recovery is attributed to immediate and successful CPR, young age, good cardiac function, and early ECMO therapy. It has been recently reported that early initiation of ECMO may lead to positive outcomes.^[20]^ Patient selection, monitoring for early detection of stroke and intracranial bleeding, and timing of ECMO are important factors influencing improvement in survival of trauma patients undergoing ECMO.^[19,20]^

## 4 Conclusions

Heparin-free VV-ECMO can be used as a resuscitative therapy in multiple trauma patients with critical respiratory failure accompanied by coagulopathy. Even in cases in which life-threatening hypoxemia and severe hypercapnia/acidosis last for >1 hours during CPR for cardiac arrest, VV-ECMO could be considered a potential lifesaving treatment.

## Author contributions

**Data curation:** Hyunyoung Joo.

**Supervision:** Hee Jung Baik, Heeseung Lee, Chi Hyo Kim, Rack Kyung Chung, Jong In Han, Jae Hee Woo.

**Writing – original draft:** Youn Young Lee, Hee Jung Baik.

**Writing – review & editing:** Youn Young Lee, Hee Jung Baik.
